# Transcriptome analysis of human OXR1 depleted cells reveals its role in regulating the p53 signaling pathway

**DOI:** 10.1038/srep17409

**Published:** 2015-11-30

**Authors:** Mingyi Yang, Xiaolin Lin, Alexander Rowe, Torbjørn Rognes, Lars Eide, Magnar Bjørås

**Affiliations:** 1 Department of Microbiology, Oslo University Hospital and University of Oslo, Norway; 2 Department of Medical Biochemistry, Oslo University Hospital and University of Oslo, Norway; 3 Department of Informatics, University of Oslo, Norway; 4 Department of Cancer Research and Molecular Medicine, Norwegian University of Science and Technology, Trondheim, Norway; 5 Key Laboratory of Carcinogenesis and Translational Research (Ministry of Education), Department of Breast Oncology, Peking University Cancer Hospital and Institute, Beijing, P.R. China

## Abstract

The oxidation resistance gene 1 (*OXR1*) is crucial for protecting against oxidative stress; however, its molecular function is unknown. We employed RNA sequencing to examine the role of human OXR1 for genome wide transcription regulation. In total, in non-treated and hydrogen peroxide exposed HeLa cells, OXR1 depletion resulted in down-regulation of 554 genes and up-regulation of 253 genes. These differentially expressed genes include transcription factors (i.e. *HIF1A*, *SP6, E2F8* and *TCF3*), antioxidant genes (*PRDX4*, *PTGS1* and *CYGB*) and numerous genes of the p53 signaling pathway involved in cell-cycle arrest (i.e. *cyclin D*, *CDK6* and *RPRM*) and apoptosis (i.e. *CytC* and *CASP9*). We demonstrated that OXR1 depleted cells undergo cell cycle arrest in G2/M phase during oxidative stress and increase protein expression of the apoptosis initiator protease CASP9. In summary, OXR1 may act as a sensor of cellular oxidative stress to regulate the transcriptional networks required to detoxify reactive oxygen species and modulate cell cycle and apoptosis.

Reactive oxygen species (ROS) are readily formed in aerobic organisms and constitute a heterogeneous group comprising superoxide anions, hydrogen peroxide, hydroxyl radicals and other free radicals, which differ in level between compartments inside the cell. In the cell, ROS may attack DNA causing accumulation of oxidative DNA damage and mutations, which are implicated in diseases such as cancer and neurodegeneration^1^,^2^,^3^.

Cells have evolved numerous mechanisms to counteract the toxic effects of ROS. For example, the antioxidant enzymes such as catalase (CAT), superoxide dismutase (SOD) and glutathione peroxidase (GPX) detoxify ROS, while the base excision repair (BER) pathway removes oxidized bases from DNA in both mitochondria and the nucleus^4^.

The eukaryotic oxidation resistance gene 1 (*OXR1*) is involved in protection against ROS and was identified by its ability to suppress mutagenesis in the DNA repair deficient *Escherichia coli nth mutH* mutant strain^5^,^6^. *OXR1* is a conserved gene family found in most eukaryotes including worms, insects and mammals, but is absent in prokaryotes. Mice lacking Oxr1 display cerebellar neurodegeneration, suggesting a neuroprotective role of OXR1 that is especially important for post-mitotic cells^7^. *C. elegans* OXR1 has also been shown to play a role in regulation of aging and maintenance of normal life span^8^. Recently it was reported that expression of Oxr1 extends the survival in a mouse model of amyotrophic lateral sclerosis^9^. However, the molecular mechanism underlying oxidative stress protection is not clear.

Previously, we demonstrated that human *OXR1 (hOXR1*) protected cells against oxidative stress by increasing expression of the antioxidant genes heme oxygenase 1 (*HMOX1* or *HO-1*) and *glutathine peroxidase* 2 (*GPX2)* via the p21 (*cyclin-dependent kinase inhibitor 1A, CDKN1A*) signaling pathway^10^. However, the p21 pathway only partly contributes to the anti-oxidation function of OXR1, suggesting that hOXR1 is important for other stress response pathways in the cell. In this paper, we use RNA sequencing (RNA-seq) to examine the role of hOXR1 in genome-wide transcription regulation including the early oxidative stress response genes, highlighting its importance in global gene expressions. We demonstrate that hOXR1 regulates cell cycle and apoptosis via the p53 signaling pathway.

## Results

### Transcriptome analysis of *hOXR1* depleted HeLa cells by RNA sequencing

To investigate the genome-wide effect of hOXR1 on gene expression regulation during oxidative stress, we compared control HeLa cells to hOXR1-depleted HeLa cells before and after exposure to hydrogen peroxide (H_2_O_2_). We have previously shown that the viability of OXR1 depleted HeLa cells exposed to 0.5 mM H_2_O_2_ for 1 h was about 90%^10^. To examine the impact of OXR1 on the early oxidative stress response, 2 days after siRNA transfection, the HeLa cells were treated with hydrogen peroxide at 0.5 mM for 1 h and harvested cells immediately without recovery (R0h). The cells were transfected with control siRNA (siCon) or human *OXR1* siRNA (siOXR1) targeting exon 19, which is common in all isoforms (*OXR1 A-D*)^10^. As previously shown hOXR1 protein expression is efficiently knocked-down by this siRNA^10^. Two days after siRNA transfection, the cells were either harvested directly (siCon_NT, siOXR1_NT) or exposed to 0.5 mM H_2_O_2_ for 1 h and then harvested immediately without recovery (siCon_R0h, siOXR1_R0h). Total RNA was isolated, sequenced and analyzed by RNA sequencing on an Illumina HiSeq2000 platform (see methods) (Table 1). For all of the samples, the sequencing resulted in more than 27 million clean reads after removing low quality reads and adaptors. The reads mapped to more than 84% of the reference genome, in which more than 70% of the reads showed unique/complete match. Further, more than 73% of the reads mapped to reference genes, in which more than 69% of the reads showed complete match. 11000 genes showed more than 50% sequence coverage.

**Figure 1:**
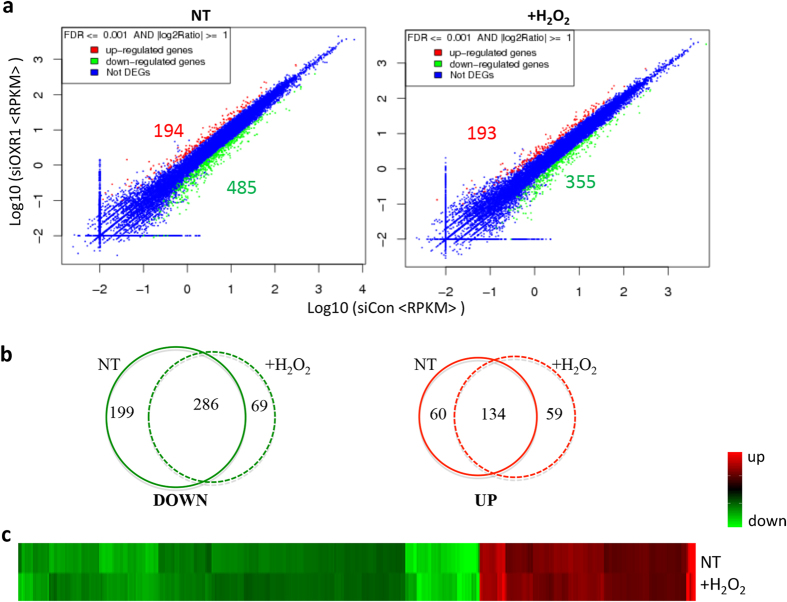
The differential expression profile in hOXR1 depleted HeLa cells. The HeLa cells were transfected with control siRNA (siCon) or hOXR1 siRNA (siOXR1) and, split into six-well plate after one day. One day later cells were treated without (NT) or with 0.5 mM H_2_O_2_ for 1 h. Cells were harvested immediately without recovery (R0h). The total RNA was isolated and analyzed by RNA-seq. (**a**) Scattered plot of hOXR1-depleted cells show differentially expressed genes without (NT) or with peroxide treatment (+H_2_O_2_). (**b**) Venn diagram analysis of common/unique down- and up-regulated genes in hOXR1 depleted cells without (NT) or with peroxide treatment (+H_2_O_2_). (**c**) Heatmap showing the overview of total DEGs caused by hOXR1 depletion. Red: up regulation; green: down regulation; black: no change.

Differentially expressed genes in *hOXR1* depleted cells. By comparing the RNA sequencing results from hOXR1 depleted cells and control cells we identified 807 differentially expressed genes (DEGs), in which 554 genes are down- regulated and 253 genes are up-regulated (Fig. 1). In non-treated hOXR1 depleted cells, we identified 485 down-regulated genes and 194 up-regulated genes as compared to the control cells (Fig. 1a) (Supplementary Table S2). After H_2_O_2_ treatment, we find 355 down-regulated genes and 193 up-regulated genes (Fig. 1a and Supplementary Table S3). Notably, comparing DEGs before and after treatment showed that 286 genes (51%) and 134 genes (53%) of the down- and up-regulated DEGs, respectively, were similarly regulated under both conditions (Fig. 1b,c). All together, these data suggest that hOXR1 has an important role in transcriptional regulation of numerous genes under normal physiology and during oxidative stress.

### Gene Ontology and pathway analysis of DEGs

**Figure 2:**
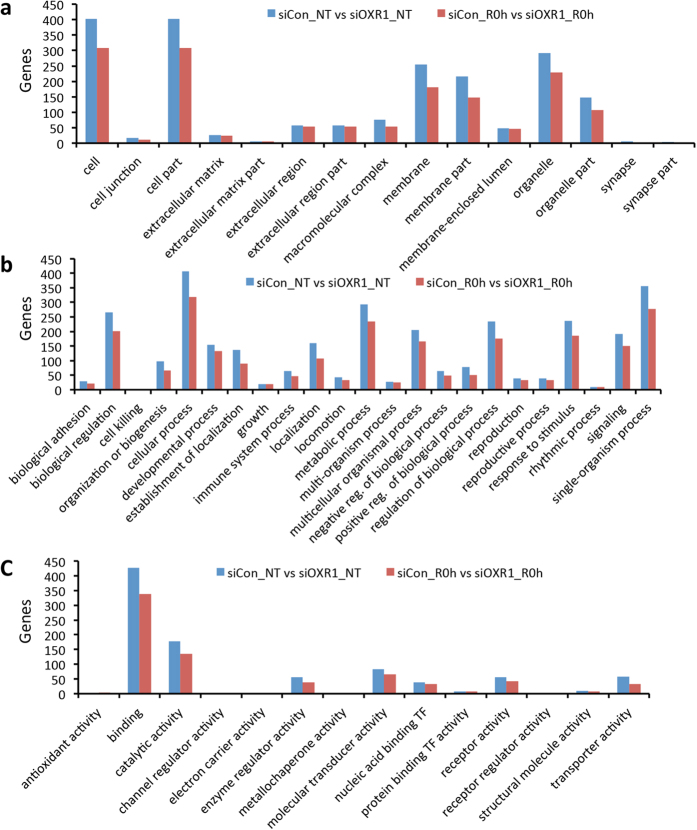
Gene ontology (GO) analysis of the deferentially expressed genes in hOXR1 depleted HeLa cells. The number of DEGs in each category was compared between non-treated (NT) and H_2_O_2_ treated (R0h) HeLa cells. (**a**) Cellular components. (**b**) Biochemical processes. (**c**) Molecular function.

Next, we performed Gene Ontology (GO) analysis of the DEGs. The GO covers three domains: cellular components, biological processes and molecular functions. A large percentage of the DEGs are associated to the membrane and organelle categories (Fig. 2a). Among the biological processes, the largest clusters include biological regulation, cellular processes, metabolic processes and response to stimulus and signaling (Fig. 2b). The hOXR1-affected transcriptome may imply a molecular function in binding, catalytic activities, enzyme regulators, molecular transducers, nucleic acid binding, receptor activities and transporter activities (Fig. 2c). Previously, we have identified two hOXR1-regulated antioxidant genes (*GPX2* and *HO-1*)^10^. In this study, three additional genes are identified in the cluster of “antioxidant activity”: *peroxiredoxin 4* (*PRDX4*), *prostaglandin-endoperoxide synthase* 1(*PTGS1*) and *cytoglobin* (*CYGB*). CYGB is a hemoglobin induced protein expressed in response to oxidative stress and involved in protection against oxidative stress induced apoptosis and cell death by scavenging ROS^11^,^12^. Notably, depletion of *hOXR1* caused down-regulation of *CYGB* and *PTGS1* as well as *HO-*1 under both oxidative stress and normal physiology (Supplementary Fig. S5).

The GO gene enrichment analysis showed several biological processes significantly affected by hOXR1 depletion in non-treated cells, including responses to hormone stimulus (41 out of 499 genes, 8.2%) and regulation of cell proliferation (28 out of 499, 5.6%) (Supplementary Table S4). Previously we showed that hOXR1 depleted HeLa cells exhibit higher cellular ROS level even before hydrogen peroxide treatment^10^. Notably, here we identified 6 DEGs belonging to the ROS response genes including *Neuronal pentraxin receptor* (*NPTXR*), *FOS-like antigen* 1(*FOSL1*), *FBJ murine osteosarcoma viral oncogene homolog* (*FOS*), *HO-1* (or *HMOX1*), *SRC proto-oncogene* (*SRC*) and *Aquaporin 1* (*AQP1*).

In the molecular function category, the top 3 enriched clusters were phospholipase C activity (7 genes), growth factor binding and phospholipase activity in non-treated cells, while the top clusters include growth factor binding, metal ion binding and activin receptor activity under H_2_O_2_ induced oxidative stress (Supplementary Table S5).

### HOXR1 regulates several branches of the p53 pathway

**Figure 3:**
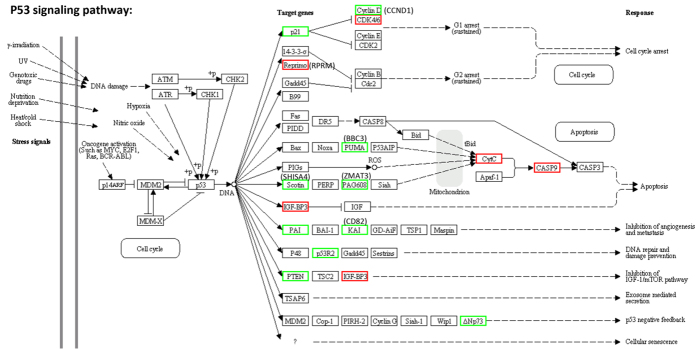
Gene expression for a subset of genes in the p53 signaling pathway was impaired by hOXR1 depletion under physical conditions. The p53 pathway is adapted from KEGG database. The up-regulated genes are labeled in red, while the down-regulated genes are labeled in green.

The Kyoto Encyclopedia of genes and Genomes (KEGG)^13^ pathway enrichment analysis showed that various pathways were affected by hOXR1 depletion in HeLa cells. In non-treated cells, the top 10 pathways include p53 signaling (Fig. 3), arachidonic acid metabolism and ECM (the extracellular matrix)-receptor interaction; while under oxidative stress, the top 10 pathways also contain p53 signaling pathway and arachidonic acid metabolism, but not ECM-receptor interaction (Supplementary Table S6). In addition, several other signaling pathways are also differentially regulated after hOXR1 depletion, such as the Hedgehog signaling, Nod (nucleotide-binding oligomerization domain containing) -like receptor signaling pathway (Supplementary Fig. S1), MAPK (mitogen-activated protein kinase) signaling, Notch signaling pathway, PPAR (peroxisome proliferator-activated receptor) signaling pathway and ErbB (epidermal growth factor receptor) signaling.

From the transcriptome analyses, the p53 signaling pathway is identified as the most frequent pathway affected by hOXR1 depletion both under normal conditions and after H_2_O_2_ exposure. Totally, about 143 genes are known to be involved in this pathway, of which 18 genes are differentially expressed in hOXR1 depleted cells (Fig. 3). Among these DEGs, 12 genes are differentially regulated under both conditions: *BBC3* (*BCL2 binding component 3*), *CASP9* (*caspase 9*), *CCND1* (*cyclin D1), CD82, CDK6* (*cyclin-dependent kinase 6*)*, CDKN1A, IGFBP3* (*insulin-like growth factor binding protein 3*), *RPRM* (*reprimo*, TP53 dependent G2 arrest mediator candidate), *THBS1* (*thrombospondin 1*), *TMEM59L* (*transmembrane protein 59-like*)*, TNS1* (*tensin 1*)*, ZMAT3* (*zinc finger matrin-type 3*); while four genes are affected only in non-treated cells: *CYCS* (*cytochrome c*, or *CytC*)*, RRM2B* (*ribonucleotide reductase M2 B*), *SERPINE1* (*serpin peptidase inhibitor, clade E, member 1*) *and TP73* (*tumor protein p73*). Two genes are differentially regulated only after H_2_O_2_ treatment: *IGFBP6* (*insulin-like growth factor binding protein 6*) *and SESN1* (*sestrin 1*).

**Figure 4:**
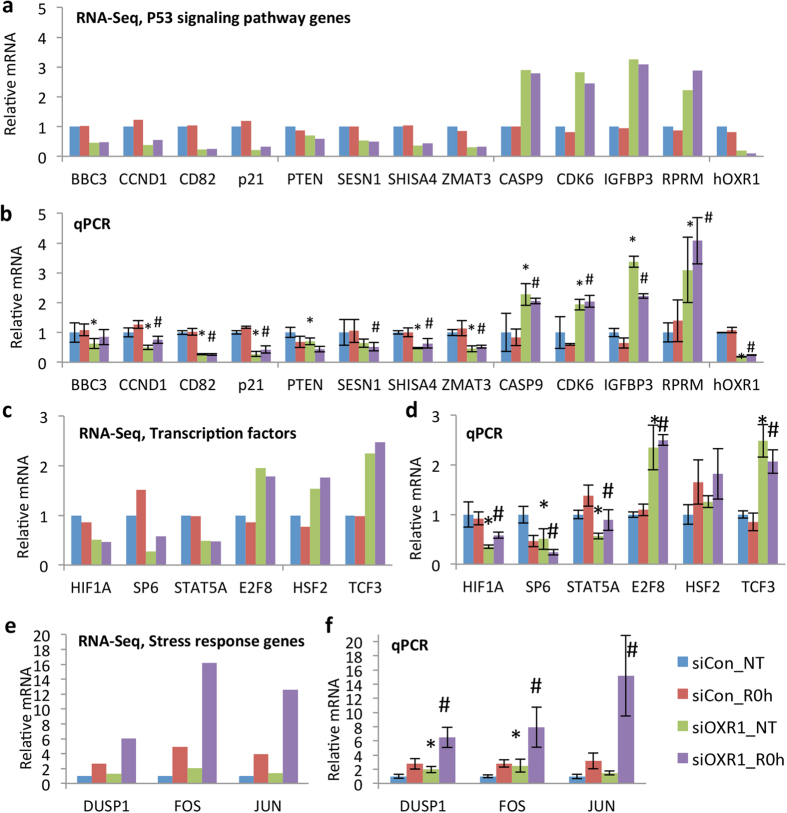
Validation of RNA-seq data by qPCR. (**a,b**) Genes involved in p53 signaling pathway. (**c,d**) Transcription factors. (**e,f**) Early stress response genes. (**a,c,e**) Data extracted from RNA-seq. (**b,d,f**) The mRNA levels of the same set of genes were measured by qPCR. The mRNA level was presented as the fold change as compared to non-treated control cells. The standard deviation was calculated from 4 cDNA samples measured in duplicate. siCon: control siRNA; siOXR1: hOXR1 siRNA;. NT: non-treatment; R0h: cells treated with H_2_O_2_ 0.5 mM for 1 h and harvested immediately without recovery. *p < 0.01 compared to siCon_NT; #p < 0.01 compared to siCon_R0h.

In order to verify the role of hOXR1 in gene expression regulation of the p53 signaling pathway, we measured mRNA levels of the differentially expressed genes in the p53 pathway using Real-Time quantitative PCR (qPCR). Consistent with the RNA-seq data, the qPCR data showed significant differential expression of all genes tested (Fig. 4a,b). For *hOXR1*, its expression in mRNA level was significantly reduced in hOXR1 siRNA transfected cells (less than 10% expression as compared to control cells (siCon) (Fig. 4a,b).

The HeLa cells contain human papilloma viruses (HPV), which express the p53-suppressive E6 protein^14^,^15^. To clarify if OXR1 siRNA interferes with p53 levels by altering the E6 levels, we performed Western blot to measure the p53 protein expression in HeLa and in an E6-negative U2OS cell line. As expected, the level of p53 protein was significantly lower in HeLa as compared to U2OS cells. The p53 protein level was independent of OXR1 in U2OS, while a slight reduction of p53 occurred in OXR1 deficient Hela cells (Supplementary Fig. S2). We compared the expression of p53-related genes upon OXR1 knockdown in the E6-negative cell line U2OS. Overall, the majority of p53 pathway genes (10 of 11 tested genes) were differentially expressed by OXR1 depletion in U2OS cells. Among these genes, 8 genes shared the same regulation pattern as HeLa (Supplementary Fig. S3), suggesting that the impact of OXR1 on p53 pathway regulation is independent of E6 protein.

### HOXR1 depletion alters expressions of transcription factors responding to cellular stress

Numerous transcription factors (TFs) are known to be involved in transcriptional networks responding to cellular stress. A total of 52 TFs were differentially expressed in *hOXR1* knockdown cells as compared to control cells (Supplementary Table S7). Further, Venn analysis showed that 20 of the 52 TFs were differentially expressed only in non-treated cells, while 14 of the TFs appeared only after hydrogen peroxide induced stress including *JUN* (*jun proto-oncogene*)*, NR4A1* (*nuclear receptor subfamily 4, group A, member 1*), *NR4A2* (*nuclear receptor subfamily 4, group A, member 2*) and *E2F8* (*E2F transcription factor 8*). 18 of the differentially expressed TFs were regulated under both conditions. Six of these genes were validated by qPCR, showing the same pattern of expression as RNA-seq, in which *HIF1A* (*hypoxia inducible factor 1, alpha subunit*), *SP6* (*Sp6 transcription factor*) and *STAT5A* (*signal transducer and activator of transcription 5A*) were down regulated, while *E2F8*, *HSF2* (*heat shock transcription factor 2*) and *TCF3* (*transcription factor 3*) were up regulated (Fig. 4c,d).

Next, we used the PASTAA software to search for possible upstream TFs with all DEGs from the RNA seq data^16^, suggesting 54 TFs with a possible regulatory association with the DEGs from hOXR1 depleted cells (Supplementary Table S8).

### Stress response genes differentially expressed in hOXR1 depleted cells

**Figure 5:**
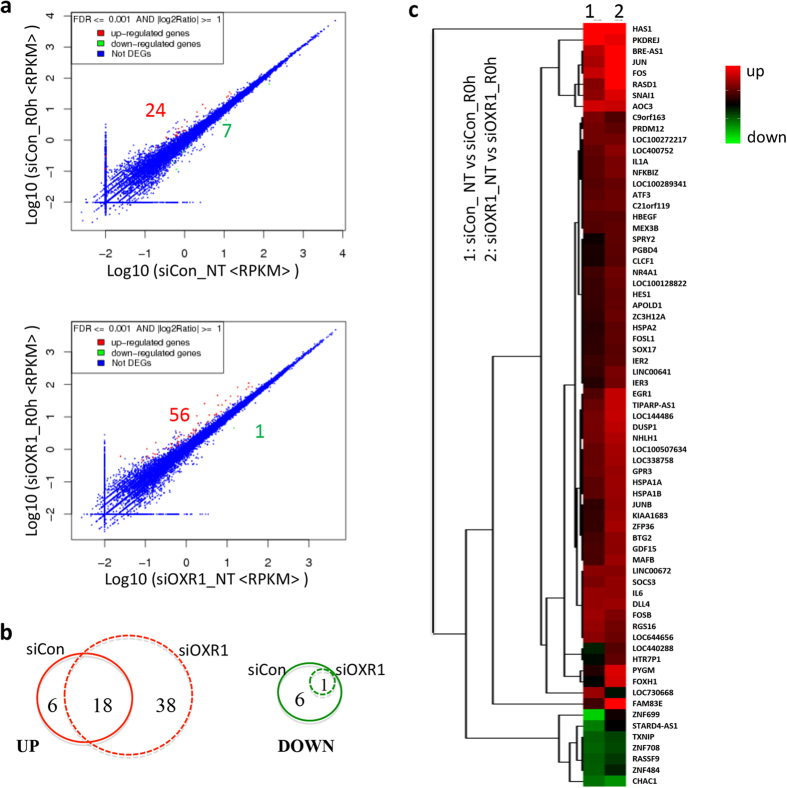
Identification of early oxidative stress response genes in control and hOXR1 depleted HeLa cells. (**a**) The differential expression profile induced by H_2_O_2_ in control siRNA (siCon_NT vs siCon_R0h) or hOXR1 siRNA transfected cells (siOXR1_NT vs siOXR1_R0h). The total numbers of up- or down-regulated genes are shown in red or green, respectively. (**b**) Venn analysis of commonly/uniquely up- or down-regulated genes in control siRNA and hOXR1 siRNA transfected cells. (**c**) Heatmap showing early oxidative stress response genes identified in control siRNA or hOXR1 siRNA transfected cells. Red: up-regulated genes; green: down-regulatied genes; black: no change; siCon: control siRNA; siOXR1: hOXR1 siRNA; NT: non-treatment; R0h: cells were treated with 0.5 mM H_2_O_2_ for1 h without recovery.

Oxidative stress induces transcriptional networks in mammalian cells. For example, exposure of the human liver HepG2 cells to hydrogen peroxide, menadione or tert-butyl hydroperoxide revealed 136 genes that are differentially expressed by all three oxidants as compared to non-treated cells^17^. In this work we identified 31 differentially expressed early stress response genes (24 up and 7 down) immediately after hydrogen peroxide exposure in control HeLa cells, while 57 genes were differentially regulated (56 up and 1 down) in hOXR1 depleted cells (Fig. 5a). Venn analysis shows that 18 DEGs are commonly regulated in both control and hOXR1 depleted cells, including transcription factors *JUN* and *FOS* (Fig. 5b,c), suggesting that hOXR1 is not necessary for up-regulation of this subset of genes during hydrogen peroxide induced stress. However, most of the genes showed a significantly stronger up-regulation in hOXR1 depleted cells as compared to control cells, including *FOS, JUN* and *DUSP1* (*dual specificity phosphatase 1*) (Figs 4e,f and 5c). Furthermore, a subset of 38 hydrogen peroxide induced genes was only found in hOXR1 depleted cells (Fig. 5b), including *IL1A* (*interleukin 1, alpha*), *HSPA2* (*heat shock 70* *kDa protein 2*), *NR4A*1 and *SPRY2* (*sprouty homolog 2*) (Fig. 5c). The proteins encoded by this subset of early stress response genes form a large protein-protein interaction network with thousands of other proteins (Supplementary Fig. S4). Thus, the increased number of early stress response genes in hOXR1 depleted cells suggests an important role for hOXR1 in regulating the early stress response during oxidative stress. GO enrichment analysis of the early stress response genes showed that the top biological processes included the stress response cluster under H_2_O_2_ induced oxidative stress in both control and hOXR1 depleted cells. Interestingly, the enriched clusters also included “RNA metabolic processes” and “regulation of transcription” in hOXR1-depleted cells, but not in control cells (Supplementary Table S9). Thus it appears that hOXR1 is crucial for balancing transcriptional networks regulating the oxidative stress response.

### hOXR1 regulates cell cycle progression

**Figure 6:**
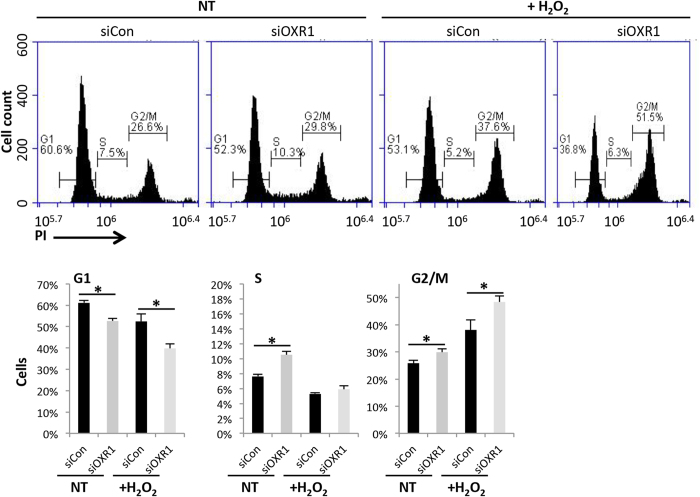
HOXR1 depletion caused cell cycle arrest in G2/M phase. The proportion of cells decreased in G1, but increased in G2/M phase in hOXR1-depleted cells (siOXR1) as compared to control (siCon). Upper panel: one representative set of flow cytometry data. The HeLa cells were exposed to 0.25 mM H_2_O_2_ for 1 h or non-treated (NT), recovered in normal medium for 24 h, fixed, stained by propidium iodide (PI) and subjected to flow cytometry. Bottom panel: Data quantification, n = 4. *p < 0.01.

In response to DNA damage the p53 signaling pathway arrests the cell cycle. In our transcriptome analysis, we find four differentially expressed genes involved in regulating cell cycle progression via the p53 pathway; *CDKN1A (p21), CCND1, CDK6* and *reprimo (RPRM)*. In hOXR1 depleted cells under both normal conditions and oxidative stress, the cell cycle inhibitor *p21* was down-regulated, the G2 arrest mediator *RPRM* was up-regulated and the G1/S transmission stimulators *CDK6* and cyclin D were up- and down-regulated, respectively. To examine the role of hOXR1 in cell cycle regulation, we measured the distribution of hOXR1 depleted HeLa and control cells in G1, S and G2/M phase by flow cytometry. First, control cells were tested at 0.25 or 0.5 mM H_2_O_2_ exposure (1 h) and 24 h recovery time, showing that 17.2% or 36.5% of the cells were enriched in G2/M, respectively. It thus appears that the cells arrest in G2/M in a dose dependent manner at these concentrations of H_2_O_2_. Next, we exposed hOXR1 depleted cells and control to the lowest dose of H_2_O_2_ (0.25 mM) to avoid cell death (more than 95% survival). Non-treated hOXR1depleted cells showed a significant reduction in number of cells in G1 phase, but increased number of cells in S and G2/M phases in comparison to control cells (Fig. 6). After exposure to peroxide, the cell numbers in both G1 and S phase decreased (Fig. 6). As expected, the population of cells in G2/M phase increased in both control and *hOXR1* silenced cells as compared to non-treated cells, confirming that the cells were mainly arrested in G2/M in response to hydrogen peroxide exposure. Importantly, the cell population in G1 was significantly lower in hOXR1 depleted cells as compared to control cells, while cell numbers in G2/M were significantly higher in hOXR1-depleted cells than control cells after hydrogen peroxide treatment. Thus it appears that hOXR1 plays an important role in cell cycle progression by regulating the p53 pathway via *p21*, *CCND1*, *CDK6* and *RPRM*.

### Depletion of hOXR1 causes up-regulation and activation of caspase 9

**Figure 7:**
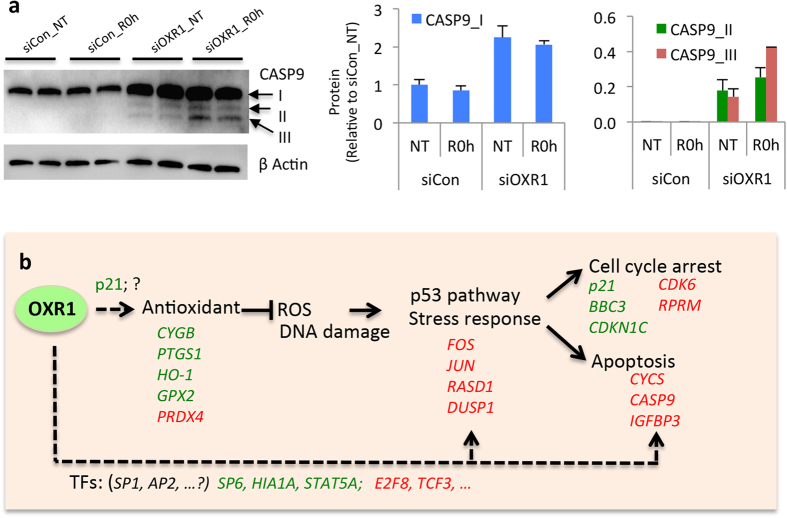
(**a**) Caspase 9 protein level increased and was partly cleaved into active forms in hOXR1 depleted HeLa. Left panel: Western blot analysis. Actin was used as internal loading control. Right panel: quantification of protein bands. siCon: control siRNA; siOXR1: hOXR1 siRNA; NT: non-treatment; R0h: cells were treated with 0.5 mM H_2_O_2_ for 1 h and harvested immediately without recovery. The quantification is the average of two independent experiments. (**b**) Model of hOXR1 mediated regulation of antioxidant defense, early stress response, cell cycle and apoptosis. HOXR1 up regulates transcription of four antioxidant genes, resulting in suppression of ROS and modulation of early stress response genes. In hOXR1 depleted cells, increased ROS leads to increased oxidative damage (i.e. DNA damage) that triggers cell cycle arrest in G2/M and apoptosis via regulation of *RPRM, CASP9* and several other genes in the p53 pathway. We postulate that hOXR1 may regulate the early stress response, cell cycle and apoptosis directly or indirectly by interaction with transcription factors such as SP1 (Sp1 transcription factor) and AP2 (activating enhancer binding protein 2). Genes labeled in red or green are up- or down-regulated, respectively, in hOXR1 depleted cells.

Previously, we showed that hOXR1 depletion induces apoptosis under oxidative stress^10^. The p53 signaling pathway plays an important role in induction of apoptosis, and three pro-apoptotic genes belonging to the p53 pathway were strongly induced by hOXR1 depletion: *CYCS, caspase 9* (*CASP9*) and *insulin-like growth factor binding protein 3* (*IGFBP3*). IGFBP3 stabilizes IGF (insulin-like growth factor) and alters its interaction with cell surface receptors. During oxidative stress, CYCS is released into the cytoplasm from mitochondria and activates Apaf1 (apoptotic peptidase activating factor 1), which in turn cleaves procaspase 9 into its active form of dimer p35/p12 or p35/p10. Another active dimer, p37/p10, is generated through cleavage of the procaspase 9 by caspase 3^18^. To examine the effect of hOXR1 depletion on the protein level, we assessed CASP9 by western analysis and found that hOXR1 depletion resulted in a significant induction as well as processing of the cellular CASP9 protein. The CASP9 antibody was raised against amino acids 100–270, which is recognizing both p37 and p35. Three bands of CASP9 were detected, of which the largest band at 47 kDa (CASP_I) is the inactive form of procaspase 9 whereas the middle band at 37 kDa (CASP_II, corresponding to p37) and the lower band at 35 kDa (CASP_III, corresponding to p35) are the active forms after cleavage (Fig. 7a). The control cells only expressed the inactive form CASP9_I, while the hOXR1 depleted cells expressed both the inactive form I and the active form II/III both in non-treated cells and after H_2_O_2_ treatment. In addition, the protein level of CASP_I increased more than 2-fold in hOXR1 depleted cells in comparison to control cells. Thus, it appears that hOXR1 regulates apoptosis via the p53 pathway by controlling *CytC* and *CASP9* expression and CASP9 activation.

## Discussion

In this report, we investigate the impact of hOXR1 on gene expression regulation by RNA-seq. Our data reveals that hOXR1 regulates the transcriptional networks required to detoxify cellular reactive oxygen species (ROS), prevent cells from oxidative DNA damage induced cell cycle arrest and apoptosis (Fig. 7b). We have previously demonstrated that the ROS level and apoptosis are increased by *OXR*1 knockdown under H_2_O_2_ stress^10^.

Recently, we demonstrated that OXR1 prevent oxidative stress induced cell apoptosis at least in part by gene expression regulation of *p21* and antioxidant gene *GPX2* and *HO-1*^10^. In the RNA-seq data, we also observed reduced transcription levels of *p21* and *HO-1* (Fig. 4a,b and Supplementary Fig. S5). The expression of *GPX2* was below detection level in the RNA-seq data. As expected, the RNA-seq showed that the *hOXR1* was largely reduced (Fig. 4a,b). In order to verify the RNA-seq data, 22 differentially expressed genes (including *hOXR1* as internal control) were checked using qPCR, showing that the relative expression level for each gene was almost the same as measured by RNA-seq (Fig. 4), except that no significant difference was found for *JUN* in non-treated cells, and for *HSF2* genes because of high variance. Thus, the RNA-seq data is reliable and of high quality.

Our transcriptome analysis shows that hOXR1 is involved in regulation of a number of pathways. Among them, the *p53* signaling pathway appears most significant in the pathway enrichment analysis. The RNA-seq data showed that hOXR1 depletion caused differential expression in a subset of genes involved in apoptosis and cell cycle regulation. Based on this data, we examined the hypothesis that hOXR1 is crucial for apoptosis and cell cycle progression. Our previous data already showed that depletion of hOXR1 increased ROS and apoptosis under oxidative stress^10^. Here we demonstrate that hOXR1 suppresses expression of the apoptosis-promoting genes *CytC* and *CASP9*. Moreover, Western blots demonstrate that hOXR1 also suppresses the level of CASP9 protein and limits cleavage into its active form. It is known that increased ROS causes the release of CytC from mitochondria to the cytosol. CytC bind and activate Apaf-1, which in turn cleaves CASP9 into its active form, triggering apoptosis^19^. Thus, hOXR1 acts to suppress oxidative stress induced apoptosis by the ROS-CytC-Caspase axis in the p53 pathway (Fig. 7b).

We observed that the HeLa cells mainly arrested in G2/M, but not in G1 or S phase after cell exposure to hydrogen peroxide (Fig. 6), which is consistent with a previous reports by Deferme *et al.*^17^. Our data revealed that the cell cycle G2 phase regulator RPRM was up-regulated in the absence of hOXR1. The flow cytometry experiments further confirmed that hOXR1 is required to regulate G2 arrest. In addition, hOXR1 is required to balance the G1/S transition, because depletion of hOXR1 caused more cells to pass from G1 into S phase under physiological conditions. Our RNA-seq data suggests that hOXR1 may balance the G1/S transition by regulating gene expression of *p21* and *CDK6*.

It is well known that cells have evolved a defense response to oxidative damage by induction of nuclear gene expression. Gene expression regulations on a genome wide scale induced by H_2_O_2_ have been studied in several human cell lines^20^,^21^,^22^,^23^. It was reported that the early stress response genes induced by H_2_O_2_ in HeLa cells included *AP-1* (*activator protein 1*) transcription factors *FOS*, *JUNB*, *BTG (B cell translocation gene)* family, *p21* and *CASP9*^23^. Among them, *FOS*, *JUNB* and *BTG2* (*BTG family, member 2)* were up-regulated immediately after exposure to H_2_O_2_ for 1 h in this study, while *p21* was up-regulated after recovering in normal medium for 3 h as shown in a previous study^10^. Furthermore, our data reveals that most of the differentially expressed early stress response genes are involved in gene expression regulation i.e. TFs such as *FOS, JUN, NFKBIZ* (*nuclear factor of kappa light polypeptide gene enhancer in B-cells inhibitor, zeta*), *ATF3* (*activating transcription factor 3*), *NR4A1* and *FOSL1*; in signal transduction i.e. *DUSP1*, *EGR1* (*early growth response 1*), *SOCS3* (*suppressor of cytokine signaling 3*) and *IL1A*; in metabolism such as *HAS1* (*hyaluronan synthase 1*), *AOC3* (*amine oxidase, copper containing 3*); in RNA interference i.e. *BRE-AS1* (*BRE antisense RNA 1*) and *TIPARP-AS1* (*TIPARP antisense RNA 1*). Vandenbroucke *et al.* performed a comparative analysis of microarray data from H_2_O_2_ induced plant, yeast and human (HeLa) cells^24^. This analysis revealed that four families of evolutionarily conserved proteins are involved in the oxidative stress response: Heat shock proteins (HSPs), small guanine triphosphate-binding proteins, Ca^2+^-dependent protein kinases, and ubiquitin-conjugating enzymes. The major oxidative stress response genes in humans belong to the families of HSPs, ATPases and D-3-phosphoglycerate dehydrogenases. The antioxidant genes such as catalase, superoxide dismutase and glutathione peroxidase were induced in yeast, but none of these were induced in human cells^24^. We also observed the induction of *HSP1A, HSP1B*, but not of ATPases or dehydrogenases. Similarly to previous reports, non-antioxidant genes were also induced in the early stress response in our experiments. However, we identified 4 antioxidant genes, which were down regulated in hOXR1 depleted cells. The basic expression levels of these genes are hOXR1 dependent, but they are not regulated under oxidative stress.

The structure of mammalian OXR1 remains to be resolved. But the 3D atomic structure of zebrafish OXR1 reveals that the conserved domain TLDc (TBC and LysM domain containing proteins) consists of two antiparallel beta-sheets forming a central beta-sandwich, surrounded by two helices and two one-turn helices. The fold shares low structural similarity to known structures. Thus, it is difficult to predict its molecular functions based on its structure at present^25^.

So far, there is no evidence that OXR1 is involved in oxidative DNA damage repair or directly detoxifying ROS. Recently, co-immunoprecipitation experiments with HA-tagged mouse Oxr1 expressed in neuronal cells identified dozens of proteins that potentially associate with OXR1, including several transcription regulators such as FUS (fused in sarcoma), CDKN2A (cyclin-dependent kinase inhibitor 2A) and TARDBP (TAR DNA-binding protein 43)^9^,^26^. We may speculate that OXR1 modulate transcriptional networks associated with oxidative stress signaling in cytosol to facilitate transcription regulation in nucleus. P53 or its up-stream factors are potential targets for interactions in cytosol that leads to transcription activation via the P53 pathway.

## Methods

### Cell culture conditions

Human tumor cell line HeLa S3 and U2OS were purchased from American Type Culture Collection (ATCC). The cells were cultured at 37 °C with 5% CO_2_ in growth medium: DMEM medium including 4.5% glucose (Biowhittaker, Cambrex Inc.) and supplemented with 10% fetal calf serum, 100 IU/ml penicillin and 100 μg/ml streptomycin. Hydrogen peroxide (H_2_O_2_ Sigma) was diluted freshly in growth medium immediately before use. Cells were treated for 1 h, following recovery in fresh medium for the indicated time.

### siRNA-mediated gene knockdown

siRNA oligos for *hOXR1* (OXR1 H6 siRNA, Cat. No. SI03246467; target sequence: 5′-AAGGAAGATTTCTTTATCCAA-3′) and negative control (Cat. No. 1027310; target sequence: 5′-AATTCTCCGAACGTGTCACGT-3′) were purchased from Qiagen, The hOXR1 siRNA maps to the 3′ end of the last exon, which is conserved in all isoforms. The siRNA oligos were transfected with Lipofectamine RNAiMAX (Invitrogen). The final concentration of siRNA oligos for transfection was 10 nM. After transfection with siRNA in 6-well plates for 24 h, the cells were seeded in 6-well plates at 20% confluence and incubated for approximately 24 h. The cells were used for further experiments including hydrogen peroxide treatment.

### RNA extraction, Illumina library preparation and sequencing

Total RNA was isolated from cell lines using RNeasy kit (Qiagen) according to the manufacturer’s instructions. RNA quality and concentration were measured using a RNA Pico chip on Agilent Bioanalyzer. In order to minimize the total number of samples for RNA sequencing, the triplicate RNA samples from each biological group was pooled together for sequencing. Library construction, sequencing with a HiSeq2000 (Illumina) instrument and the main part of bioinformatic analysis were performed through a commercially available service provided by the Beijing Genomics Institute (BGI), China. For libray prepariton, the mRNA was enriched using the oligo(dT) magnetic beads, following fragmentation (about 200 bp). Then the first strand of cDNA was synthesized using random hexamer-primer. The second strand was further synthesized in a reaction buffer including dNTPs, RNase H and DNA polymerase I. The double strand cDNA was purified with magnetic beads. Then, the 3′-end single nucleotide A (adenine) was added and adaptors were ligated to the fragments. The fragments were enriched by PCR amplification. Sequenced reads were trimmed for adaptor sequence, and masked for low-complexity or low-quality sequence. The clean reads (single-end reads) were mapped to human genome reference hg19 and reference genes (NCBI sequence database RefSeq) using SOAPaligner/SOAP2^27^. Pathway enrichment was performed by BLAST (-p blastx -e 1e−5 -m 8) using KEGG database as reference. The data was cleaned by filtering out transcripts expressed at levels below 0.5 reads per kilobase of transcript per million reads mapped (RPKM). Differential expression levels > = 2 folds change are considered as significantly different expression.

### Gene ontology analysis

The Blast2GO program^28^ (default parameters) was used to generate gene ontology (GO) annotation of differentially expressed genes (DEGs). After obtaining GO annotations for DEGs, the WEGO software^29^ was used to further perform GO functional classification and to predict pathways affected.

### Real-time qPCR

Two-steps qPCR methods were used to determine RNA levels for selected genes. First, the cDNA was generated from total RNA samples using the High-capacity cDNA reverse transcription kit from Applied Biosystem. Real-time PCR reactions were prepared using Power SYBR Green Kit including about 10 ng cDNA in each reaction, and PCR was performed in the StepOnePlus^TM^ Real-Time PCR System (Applied Biosystem) with the standard cycle conditions: 95 °C for 10 min; 40 cycles at 94 °C for 15 s and 65 °C for 30 s. Samples were measured in triplicate. *GAPDH* (*glyceraldehyde 3-phosphate dehydrogenase*) was used as an internal standard as indicated. In the RNA sequencing data, the *GAPDH* mRNA showed no significant change by *OXR1* siRNA transfection or H_2_O_2_ treatment, confirming it is stable and reliable as internal control (Supplementary Fig. S6). The expression level was calculated by the comparative cycle threshold (CT) method and represented as relative quantification (RQ). The CT for the target gene was normalized to the CT for *GAPDH* in the same sample, giving ΔCT for each sample (ΔCT = CT for target gene – CT for *GAPDH*). The average ΔCT from control samples was used as the ΔCT calibrator for the calculation of ΔΔCT. RQ is defined as 2^−ΔΔCT^, where ΔΔCT = ΔCT sample − ΔCT calibrator. A standard curve for each target gene was generated to confirm that the real PCR reactions were run in the linear range. The primer pairs for each gene are in Supplementary Table S1.

### Western blot and antibodies

The cell pellets were suspended in lysis buffer (10 mM Tris pH 7.5; 150 mM NaCl; 0.5 mM EDTA; 0.5% NP-40) for 15 min at 4 °C and centrifuged at 13000 RPM 15 min. The soluble protein extracts were separated by SDS-PAGE and transferred on a PVDF membrane (Trans-blot Turbo mini Format 0.2 μm PVDF, cat.170–4156 by BIO-RAD) using a BioRad Trans-blot instrument. The membrane was blocked with 5% milk in TBS-T buffer (50 mM Tris pH7.5; 150 mM NaCl; 0.05% Tween-20) overnight at 4 °C following incubation with the indicated primary antibody overnight at 4 °C, washed 3 times with TBS-T buffer, incubated with secondary antibody IgG-HRP at 1:20000 dilution in TBS-T buffer (cat. No.: NA934V for anti-rabbit; NA931V for anti-mouse by GE Healthcare). After washing 3 times with TBS-T buffer, the membrane was developed with ECL substrate (SuperSignal West Femto Maximum Sensitivity Substrate, by Thermo Scientific) and the signal was detected by a BIO-RAD Fluorescent Imager, following quantification by Image Lab software. The following antibodies were used in this study: caspase-9 p35 (A-9) (cat. sc-133109 by Santa Cruz biotechnology) diluted at 1: 1000 in TBS-T including 3% milk; β Actin (AC-15) antibody (cat. sc-69879 by Santa Cruz biotechnology) diluted 1:2000 in TBS-T buffer. By comparing the protein levels between the commonly used loading control β Actin and GAPDH, we showed that both of them were stably expressed in HeLa cells during *OXR1* knockdown and H_2_O_2_ treatment (Supplementary Fig. S7), confirming they are both reliable as loading controls. β Actin was used as loading control in this study.

### Flow cytometry

The cell cycle was measured by propidium iodide (PI) staining based on the protocol described by Krishan A. *et al.*^30^. The cells were seeded on a 6 cm dish at 20% confluence and cultured overnight. On the second day, the cells were exposed to hydrogen peroxide (diluted to indicated concentration with growth medium) for 1 h and recovered in growth medium for 24 h. The cells were washed once with PBS, trypsinized with 0.7 ml trypsin for 3–5 min following inactivation of trypsin by addition of 0.7 ml 10% serum. The cells were transferred to a 1.5 ml eppendorf tube and centrifuged at 3000 RPM for 5 min. The cell pellets were washed with 1 ml PBS, centrifuged at 3000 RPM for 5 min, re-suspended in 1 ml 70% ethanol (cold), stored at −20 °C over night or up to one week. The cell pellets were collected by spinning at 5000 RPM 3 min, resuspended in 0.5 ml PI staining solution (0.1% Triton X-100; 4 mM Na citrate; 50 μg/ml PI) including 100 μg/ml RNaseA, and incubated for 30 min at 37 °C in the dark. Cell cycle distribution was measured by flow cytometry (BD Accuri C6, BD Biosciences) immediately by counting a minimum of 10 000 cells. The data was quantified using the BD Accuri C6 sample software.

### Statistical analysis

Statistical analysis was performed by calculating the P-value with t-tests in two tails assuming equal variances between two groups. A confidence level of 95% (P < 0.05) was considered statistically significant difference.

### Data accessibility

The sequence data from this study have been submitted to the Gene Expression Omnibus (GEO) database (http://www.ncbi.nlm.nih.gov/geo) under accession number GSE65949.

## Additional Information

**How to cite this article**: Yang, M. *et al.* Transcriptome analysis of human OXR1 depleted cells reveals its role in regulating the p53 signaling pathway. *Sci. Rep.* **5**, 17409; doi: 10.1038/srep17409 (2015).

## Supplementary Material

Supplementary Information

Dataset 1 (Supplementary table S2)

Dataset 2 (Supplementary table S3)

Dataset 3 (Supplementary table S8)

## Figures and Tables

**Table 1:** Summary of RNA sequencing data.

Sample	Total reads	Reads mapped to genome		Mapped genes at coverage >50%	
		Reads	Percentage	Genes	Percentage
siCon_NT	29,196,944	24,995,211	85.6%	11279	67.0%
siOXR1_NT	27,803,703	23,716,261	85.3%	11096	65.9%
siCon_R0h	29,909,352	25,425,525	85.0%	11329	67.3%
siOXR1_R0h	29,623,049	25,055,148	84.6%	11200	66.5%
