# Supplementary material for: Transcriptome analysis of human OXR1 depleted cells reveals its role in regulating the p53 signaling pathway
Source: Sci Rep. 2015 Nov 30;5:17409. doi: 10.1038/srep17409 (PMC4663793; doi:10.1038/srep17409)
Supplement: Supplementary Information [file srep17409-s1.doc]

**Transcriptome analysis of human OXR1 depleted cells reveals its role in regulating the p53 signaling pathway**

Mingyi Yang^1,2^, Xiaolin Lin^2^, Alexander Rowe^2^, Torbjørn Rognes^1,3^, Lars Eide ^2^, Magnar Bjørås^1,2,4§^

**SUPPLEMENTARY FIGURES**

**Figure S1.** hOXR1 involved in regulation Hedgehog pathway. The OXR1 depletion induced up-regulation genes are labeled in red, while the down-regulation genes are labeled in green.

**
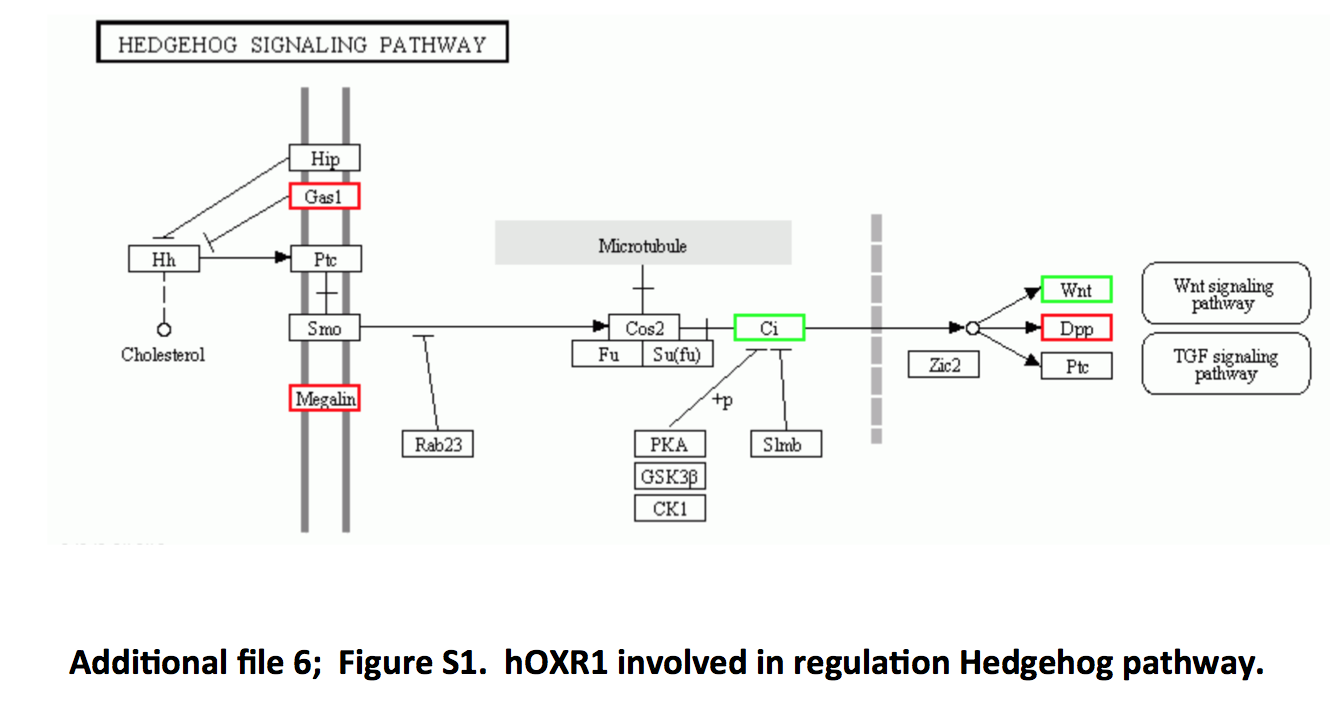
**

**Figure S2.** The p53 protein was qunatified in OXR1 depleted HeLa and U2OS cells. The cells were collected two days after transfection with control siRNA (siCon) or OXR1 siRNA (siOXR1), subsequently lysed by RIPA buffer and analyzed by Western blot. P53 was detected with a mouse monoclonal p53 antibody (DO-1, sc-126 by Santa Cruz). β Actin was used as internal loading control. Upp pannel: protein quantificaiton in three experiments. Bottom pannel: one represented Western blot experiment.


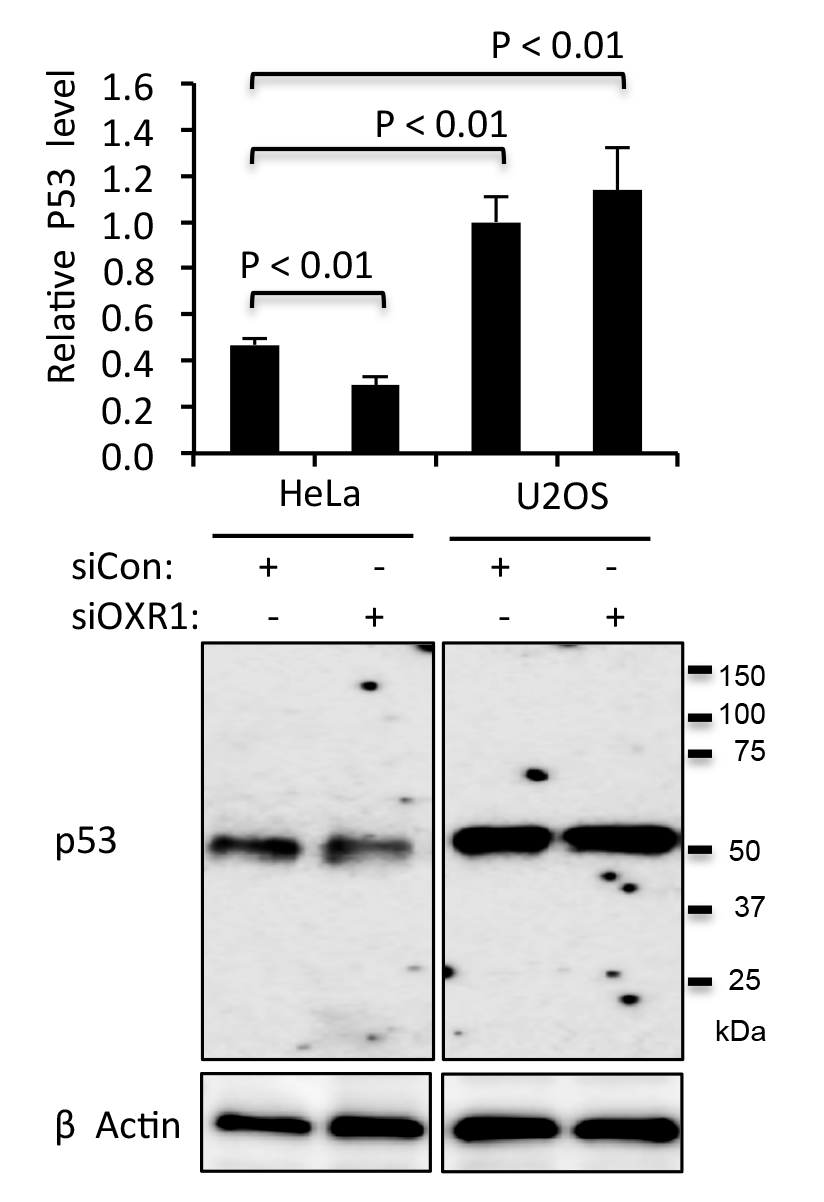


**Figure S3.** A subset of p53 pathway genes was differentially expressed by OXR1 knockdown in U2OS cells. The gene expression in mRNA level was measured by qPCR. The mRNA level was presented as the fold change as compared to non-treated control cells. The standard deviation was calculated from 4 cDNA samples measured in duplicate. siCon: control siRNA; siOXR1: hOXR1 siRNA;. NT: non-treatment; R0h: cells treated with H_2_O_2_ 0.5 mM for1 h and harvested immediately without recovery. * p < 0.01 compared to siCon_NT; # p < 0.01 compared to siCon_R0h.

**
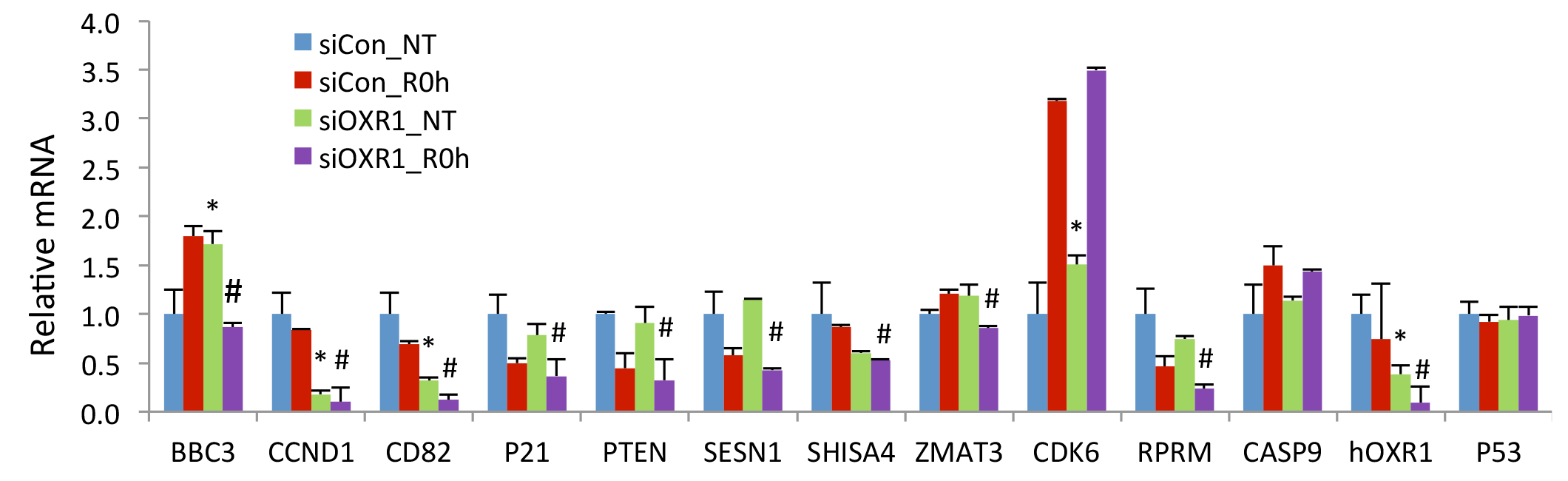
**

**Figure S4.** Comparison of Protein Interaction Networks of H_2_O_2_ induced DEGs in control and hOXR1 depleted HeLa cells.

**
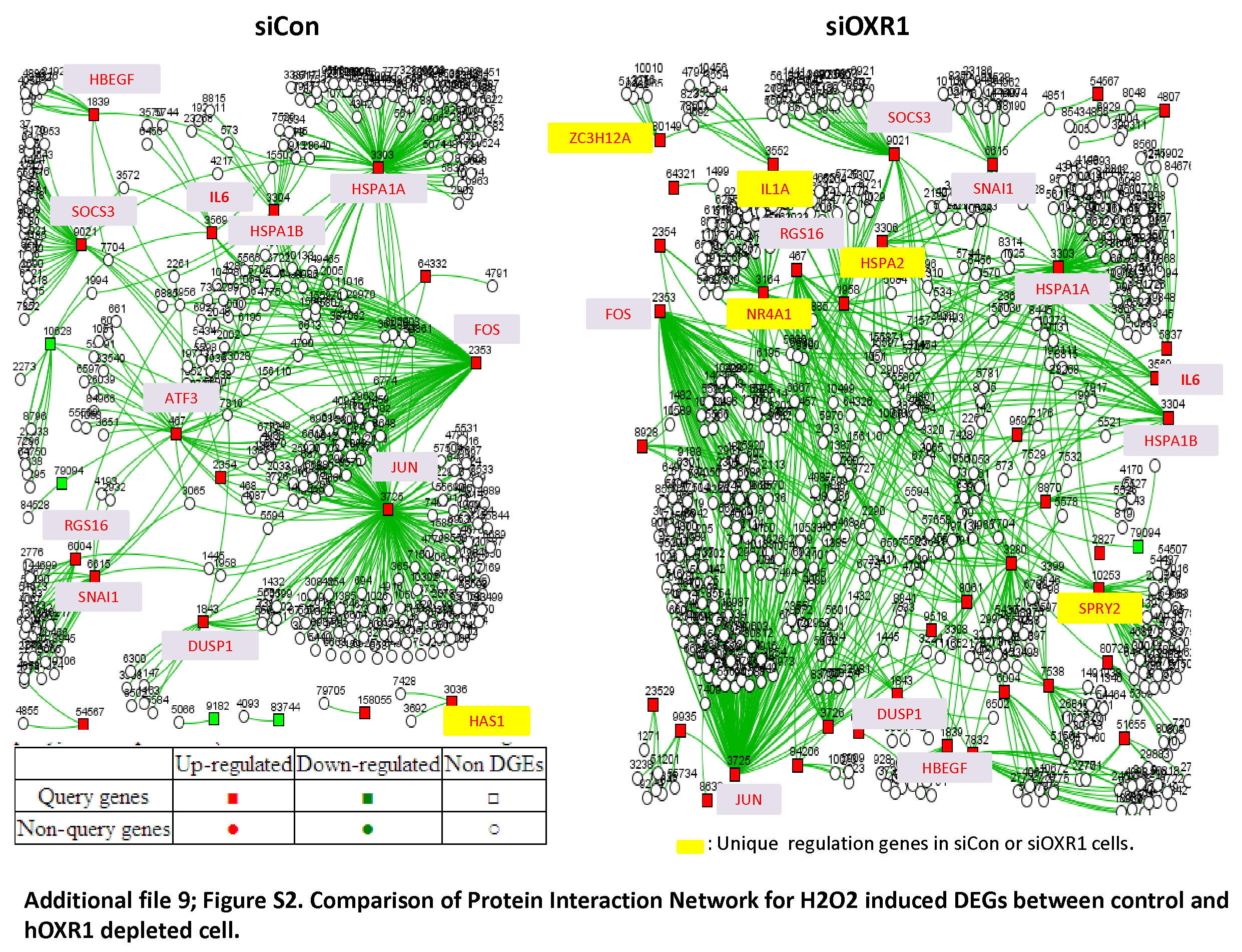
**

**Figure S5.** Differentially expressed antioxidant genes between hOXR1 depleted HeLa cells and control cells in RNA-seq data.


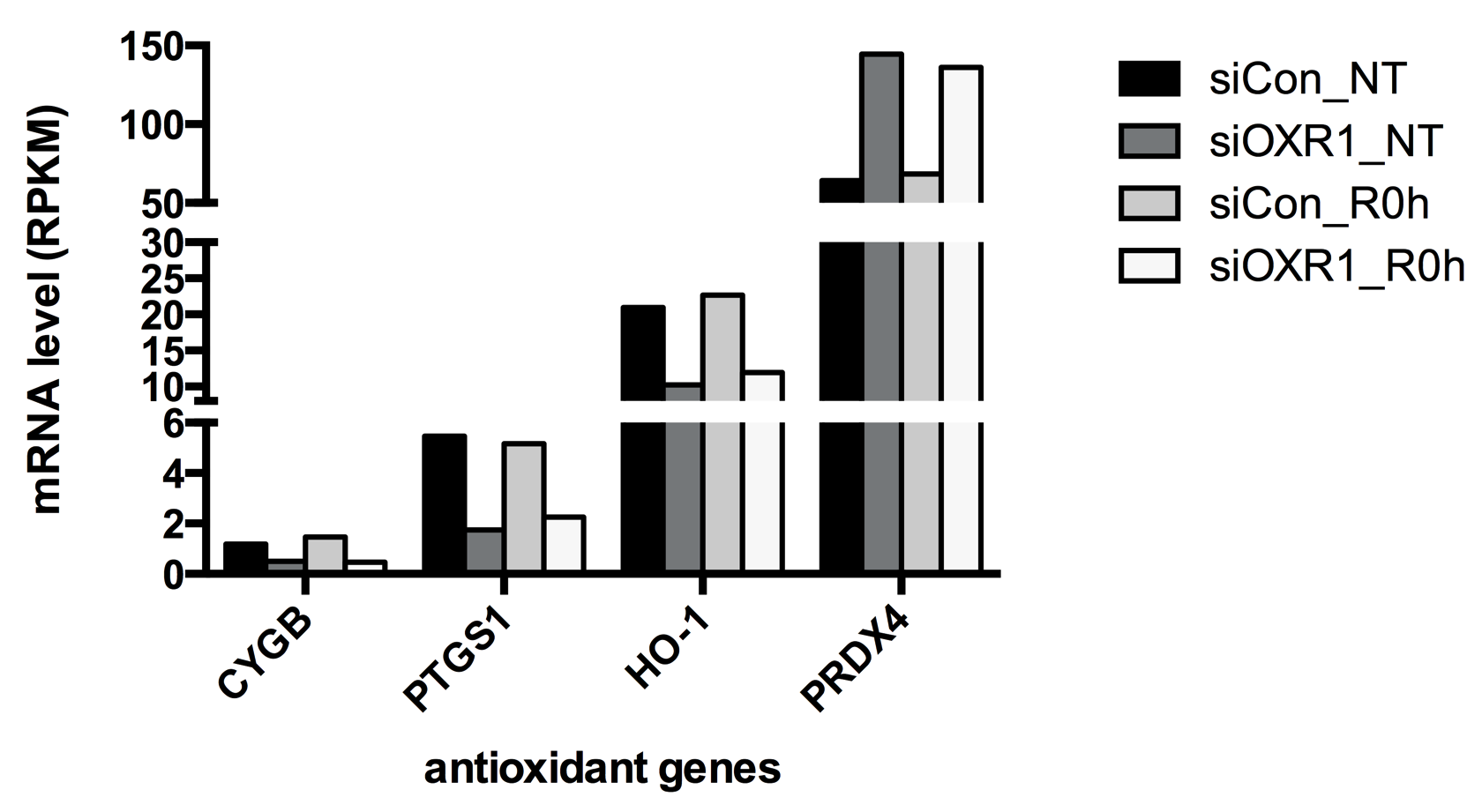


**Figure S6.**  The GAPDH mRNA level from RNA sequencing data. The number above column indicates the percentage change of GAPDH relative to reference sample (R).


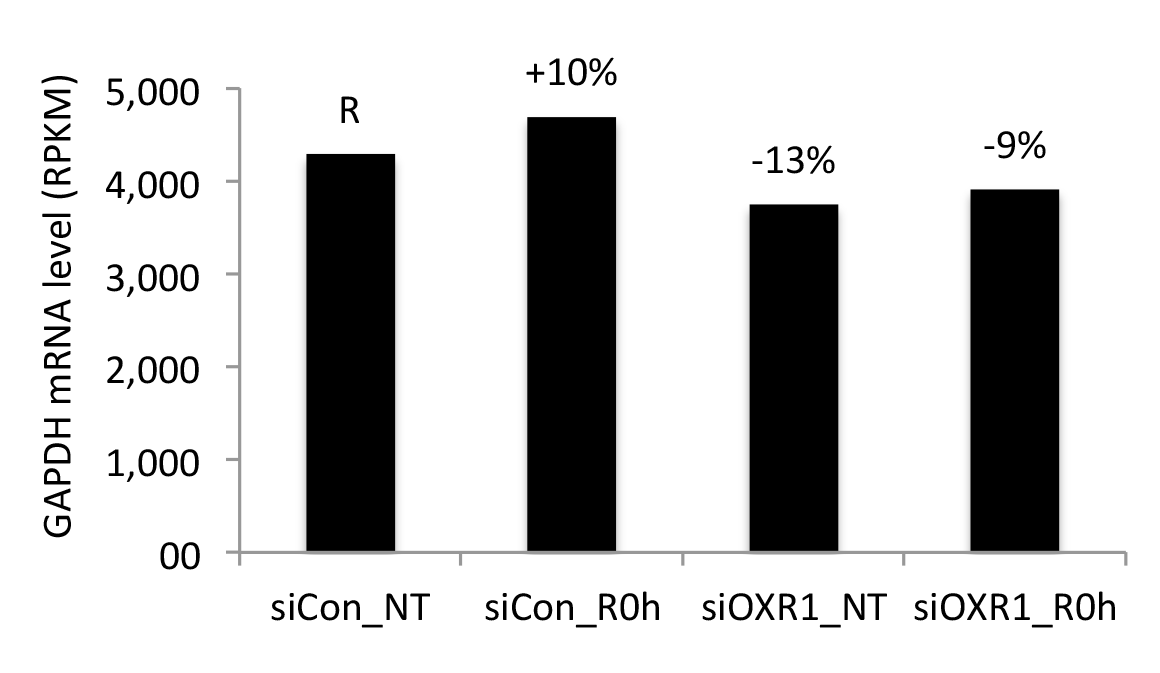


**Figure S7.** The protein level of loading control β Actin is compared with GAPDH expression in HeLa cells. The control siRNA (siCon) or OXR1 siRNA (siOXR1) transfected cells were non-treated (NT) or treated with 0.5 mM H_2_O_2_ for 1 h, collected immediately without recovery (R0h), following to be lysated with RIPA buffer for Western blot. GAPDH was detected with a mouse monoclonal antibody (ab9482 by abcam). The ratio of β Actin / GAPDH in protein level is neutralized as 1:1 in the sample siCon_NT.


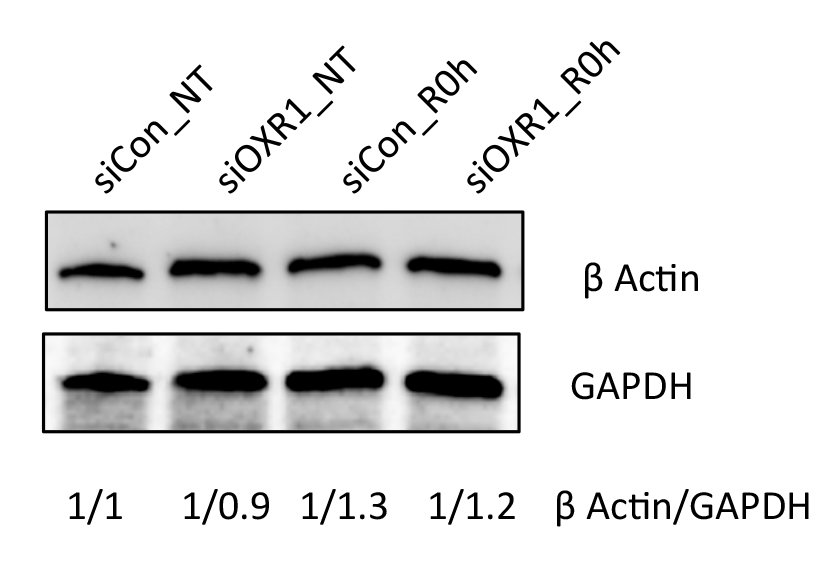


**SUPPLEMENTARY TABLES**

**## Table S2-3, 8: See Excel file 1-3.**

# Table S1. Primer pairs used in qPCR

| **Name** | **Cat. No.** | **Sequence (from 5' to 3')** |
| --- | --- | --- |
| **p53 pathway:** |  |  |
| BBC3_F | 17826 | GAGCGGCGGAGACAAGAG |
| BBC3_R | 17827 | TAAGGGCAGGAGTCCCATGA |
| CASP9_F | 17840 | TCTACGGCACAGATGGATGC |
| CASP9_R | 17841 | CATGGTCTTTCTGCTCCCCA |
| CCND1_F | 17824 | ATCAAGTGTGACCCGGACTG |
| CCND1_R | 17825 | CTTGGGGTCCATGTTCTGCT |
| CD82_F | 17832 | GTGGAAGGAAGCTCCAGGAC |
| CD82_R | 17833 | CACTGCGCCCAGGATAAAGA |
| CDK6_F | 17842 | GGCTCTAACCTCAGTGGTCG |
| CDK6_R | 17843 | GCAGCCAACACTCCAGAGAT |
| hOXR1_F | 11049 | TTATGGTACTGGAGAGACCTTTGTTTT |
| hOXR1_R | 11050 | AAAACATATTATCTCCTGTCCACTTAAAGAC |
| IGFBP3_F | 17838 | GCCAGCTCCAGGAAATGCTA |
| IGFBP3_R | 17839 | GGGGTGGAACTTGGGATCAG |
| p21_F | 11053 | GGCGGGCTGCATCCA |
| p21_R | 11054 | AGTGGTGTCTCGGTGACAAAGTC |
| PTEN_F | 17836 | TGCAGAGTTGCACAATATCCT |
| PTEN_R | 17837 | CACCAGTTCGTCCCTTTCCA |
| RPRM_F | 17844 | GGGGTCTCACAAATCCGTGT |
| RPRM_R | 17845 | CTGTAACTCCTCAGGCAGGC |
| SESN1_F | 17834 | GGGAGTGAAGACGCACAGAT |
| SESN1_R | 17835 | CTTGCCGCAGCCATTATTCC |
| SHISA4_F | 17828 | CCCATTTGAAGGCCAGGAGAT |
| SHISA4_R | 17829 | ATATGAAGCCAGGCTGTGGG |
| ZMAT3_F | 17830 | TCATTCTCGGAATCCTCAGAGC |
| ZMAT3_R | 17831 | CGAGAGCGGGGATTGAAGTA |
|  |  |  |
| **Transcription factors:** | |  |
| E2F8_F | 17856 | TGGAATTTCGGGCAGCTTCT |
| E2F8_R | 17857 | TCTGAGGCGTTGACACCAAA |
| HIF1A_F | 17846 | TCCAAGAAGCCCTAACGTGT |
| HIF1A_R | 17847 | GCAGTCTACATGCTAAATAATTCCT |
| HSF2_F | 17854 | AACGTAAAAGGCCTCTACTTCTA |
| HSF2_R | 17855 | TTGAATGCATGAAACACTGCCA |
| SP6_F | 17848 | AAAAGCTTCTGAGGCCGTGA |
| SP6_R | 17849 | CTCAGACGGGACCAGTCCAT |
| STAT5A_F | 17850 | GCTCCAGTGCAGCTCTCC |
| STAT5A_R | 17851 | CCTCAGGCTCTCCTGGTACT |
| TCF3_F | 17852 | ACTCCTACAGTGGGCTAGGG |
| TCF3_R | 17853 | CTTTCTCCTCCAGGGACAGC |
|  |  |  |
| **H_2_O_2_ induced genes:** | |  |
| FOS_F | 17858 | GGGGCAAGGTGGAACAGTTA |
| FOS_R | 17859 | GTCTGTCTCCGCTTGGAGTG |
| JUN_F | 17860 | GTGCCGAAAAAGGAAGCTGG |
| JUN_R | 17861 | CTGCGTTAGCATGAGTTGGC |
| DUSP1_F | 17862 | GGATACGAAGCGTTTTCGGC |
| DUSP1_R | 17863 | GGCCACCCTGATCGTAGAGT |
|  |  |  |
| **Internal control:** |  |  |
| GAPDH_F | 15312 | CCACATCGCTCAGACACCAT |
| GAPDH_R | 15313 | GCGCCCAATACGACCAAAT |

Notice: “_F”: forward primer; “_R”: Reverse primer.

**Table S4. The GO enrichment analysis of DEGs in the biological process category.**

**SiCon_NT vs siOXR1_NT:**

| **Gene Ontology term** | **Cluster frequency** | **Genome frequency of use** | **Corrected P-value** |
| --- | --- | --- | --- |
| Response to corticosteroid stimulus | 24 out of 499 genes, 4.8% | 132 out of 14596 genes, 0.9% | 2.10E-08 |
| Response to steroid hormone stimulus | 32 out of 499 genes, 6.4% | 285 out of 14596 genes, 2.0% | 4.23E-06 |
| Icosanoid metabolic process | 12 out of 499 genes, 2.4% | 55 out of 14596 genes, 0.4% | 0.00032 |
| Unsaturated fatty acid metabolic process | 12 out of 499 genes, 2.4% | 56 out of 14596 genes, 0.4% | 0.0004 |
| Response to hormone stimulus | 41 out of 499 genes, 8.2% | 510 out of 14596 genes, 3.5% | 0.00042 |
| Response to organic substance | 61 out of 499 genes, 12.2% | 944 out of 14596 genes, 6.5% | 0.00135 |
| Response to glucocorticoid stimulus | 10 out of 499 genes, 2.0% | 43 out of 14596 genes, 0.3% | 0.00175 |
| Response to endogenous stimulus | 44 out of 499 genes, 8.8% | 610 out of 14596 genes, 4.2% | 0.00302 |
| Regulation of cell proliferation | 28 out of 499 genes, 5.6% | 314 out of 14596 genes, 2.2% | 0.00468 |

SiCon_R0h vs siOXR1_R0h:

| **Gene Ontology term** | **Cluster frequency** | **Genome frequency of use** | **Corrected P-value** |
| --- | --- | --- | --- |
| Response to corticosteroid stimulus | 18 out of 395 genes, 4.6% | 132 out of 14596 genes, 0.9% | 1.80E-05 |
| Icosanoid metabolic process | 12 out of 395 genes, 3.0% | 55 out of 14596 genes, 0.4% | 2.14E-05 |
| Unsaturated fatty acid metabolic process | 12 out of 395 genes, 3.0% | 56 out of 14596 genes, 0.4% | 2.66E-05 |
| Prostanoid metabolic process | 8 out of 395 genes, 2.0% | 26 out of 14596 genes, 0.2% | 0.00029 |
| Response to organic substance | 48 out of 395 genes, 12.2% | 944 out of 14596 genes, 6.5% | 0.01883 |
| Anatomical structure morphogenesis | 58 out of 395 genes, 14.7% | 1230 out of 14596 genes, 8.4% | 0.02217 |
| Tonic smooth muscle contraction | 3 out of 395 genes, 0.8% | 4 out of 14596 genes, 0.0% | 0.08258 |
| Regulation of reproductive process | 6 out of 395 genes, 1.5% | 28 out of 14596 genes, 0.2% | 0.09205 |
| Positive regulation of cell proliferation | 15 out of 395 genes, 3.8% | 177 out of 14596 genes, 1.2% | 0.10152 |
| Response to steroid hormone stimulus | 20 out of 395 genes, 5.1% | 285 out of 14596 genes, 2.0% | 0.10765 |

**Table S5. The GO enrichment analysis of DEGs in the molecular function category.**

SiCon_NT vs siOXR1_NT:

| **Gene Ontology term** | **Cluster frequency** | **Genome frequency of use** | **Corrected P-value** |
| --- | --- | --- | --- |
| Phospholipase C activity | 7 out of 524 genes, 1.3% | 28 out of 15165 genes, 0.2% | 0.00959 |
| Growth factor binding | 11 out of 524 genes, 2.1% | 87 out of 15165 genes, 0.6% | 0.05305 |
| Phospholipase activity | 9 out of 524 genes, 1.7% | 61 out of 15165 genes, 0.4% | 0.06189 |
| Substrate-specific transmembrane transporter activity | 49 out of 524 genes, 9.4% | 842 out of 15165 genes, 5.6% | 0.06483 |
| Enzyme regulator activity | 55 out of 524 genes, 10.5% | 985 out of 15165 genes, 6.5% | 0.07657 |
| Vitamin transporter activity | 5 out of 524 genes, 1.0% | 18 out of 15165 genes, 0.1% | 0.07692 |
| Transmembrane transporter activity | 52 out of 524 genes, 9.9% | 929 out of 15165 genes, 6.1% | 0.1064 |
| Lipase activity | 9 out of 524 genes, 1.7% | 66 out of 15165 genes, 0.4% | 0.1134 |
| Metal ion binding | 136 out of 524 genes, 26.0% | 3038 out of 15165 genes, 20.0% | 0.1323 |

SiCon_R0h vs siOXR1_R0h:

| **Gene Ontology term** | **Cluster frequency** | **Genome frequency of use** | **Corrected P-value** |
| --- | --- | --- | --- |
| Growth factor binding | 12 out of 407 genes, 2.9% | 87 out of 15165 genes, 0.6% | 0.00079 |
| Metal ion binding | 110 out of 407 genes, 27.0% | 3038 out of 15165 genes, 20.0% | 0.07936 |
| Activin receptor activity | 3 out of 407 genes, 0.7% | 7 out of 15165 genes, 0.0% | 0.14377 |
| Pattern binding | 13 out of 407 genes, 3.2% | 169 out of 15165 genes, 1.1% | 0.14987 |
| Enzyme activator activity | 20 out of 407 genes, 4.9% | 340 out of 15165 genes, 2.2% | 0.20822 |
| Polysaccharide binding | 12 out of 407 genes, 2.9% | 159 out of 15165 genes, 1.0% | 0.28246 |
| Glycosaminoglycan binding | 11 out of 407 genes, 2.7% | 142 out of 15165 genes, 0.9% | 0.36221 |
| Oxidoreductase activity, acting on the CH-OH group of donors, NAD or NADP as acceptor | 9 out of 407 genes, 2.2% | 101 out of 15165 genes, 0.7% | 0.36329 |
| Peptide binding | 12 out of 407 genes, 2.9% | 167 out of 15165 genes, 1.1% | 0.42946 |

Table S6. Top pathways affected by OXR1 depletion in HeLa cells

| ***No.*** | ***Pathway*** | ***DEGs with pathway annotation (582)*** | ***All genes with pathway annotation (17252)*** | ***Pvalue*** | ***Qvalue*** |
| --- | --- | --- | --- | --- | --- |
| **Non-treatment:** | | | | | |
| 1 | p53 signaling pathway | 16 (2.75%) | 143 (0.83%) | 2.661014e-05 | 0.005614740 |
| 2 | Dorso-ventral axis formation | 10 (1.72%) | 67 (0.39%) | 7.754004e-05 | 0.008180474 |
| 3 | Complement and coagulation cascades | 17 (2.92%) | 181 (1.05%) | 0.0001390465 | 0.009779604 |
| 4 | Small cell lung cancer | 13 (2.23%) | 143 (0.83%) | 0.001117015 | 0.047767615 |
| 5 | Arachidonic acid metabolism | 11 (1.89%) | 109 (0.63%) | 0.001131934 | 0.047767615 |
| 6 | Vibrio cholerae infection | 12 (2.06%) | 132 (0.77%) | 0.001710352 | 0.060147379 |
| 7 | ECM-receptor interaction | 19 (3.26%) | 269 (1.56%) | 0.002020939 | 0.060916876 |
| 8 | Pathways in cancer | 30 (5.15%) | 531 (3.08%) | 0.004216946 | 0.111221951 |
| 9 | Focal adhesion | 27 (4.64%) | 475 (2.75%) | 0.00595621 | 0.131513114 |
| 10 | Prion diseases | 9 (1.55%) | 99 (0.57%) | 0.006232849 | 0.131513114 |
| **After hydrogen peroxide treatment:** | |  |  |  |  |
| 1 | p53 signaling pathway | 14 (3.02%) | 143 (0.83%) | 3.200404e-05 | 0.006560828 |
| 2 | Arachidonic acid metabolism | 11 (2.37%) | 109 (0.63%) | 0.0001696294 | 0.017387013 |
| 3 | Complement and coagulation cascades | 12 (2.59%) | 181 (1.05%) | 0.003686828 | 0.251933247 |
| 4 | Systemic lupus erythematosus | 12 (2.59%) | 188 (1.09%) | 0.00498864 | 0.255667800 |
| 5 | Dorso-ventral axis formation | 6 (1.29%) | 67 (0.39%) | 0.009169813 | 0.331832329 |
| 6 | Primary bile acid biosynthesis | 4 (0.86%) | 32 (0.19%) | 0.01023332 | 0.331832329 |
| 7 | Cocaine addiction | 7 (1.51%) | 91 (0.53%) | 0.01133086 | 0.331832329 |
| 8 | Hedgehog signaling pathway | 6 (1.29%) | 73 (0.42%) | 0.01368519 | 0.350682994 |
| 9 | Pathways in cancer | 23 (4.96%) | 531 (3.08%) | 0.01735565 | 0.356512630 |
| 10 | Prion diseases | 7 (1.51%) | 99 (0.57%) | 0.01739086 | 0.356512630 |

**Table S7. Transcription factors (TFs) differentially expressed in hOXR1 depleted cells. NT: non-treated cells; R0h: the cells were exposed in 0.5 mM hydrogen peroxide 1 h and recovered 0 h.**

| **NT** | **R0h** | **common** | **unique_NT** | **unique_R0h** |
| --- | --- | --- | --- | --- |
| BTG2 | C11orf9 | CREB3L3 | BTG2 | C11orf9 |
| CREB3L3 | CREB3L3 | ETV5 | DLX3 | E2F8 |
| DLX3 | E2F8 | FOS | DLX4 | EGR1 |
| DLX4 | EGR1 | FOSB | EHF | HES6 |
| EHF | ETV5 | FOXN1 | FOXA1 | HSF2 |
| ETV5 | FOS | FOXP4 | HNF4A | JUN |
| FOS | FOSB | GLI1 | HOXA3 | LHX1 |
| FOSB | FOXN1 | KLF6 | HOXA5 | MTA1 |
| FOXA1 | FOXP4 | MAFA | HSF4 | MYT1L |
| FOXN1 | GLI1 | MXD1 | JUNB | NR4A1 |
| FOXP4 | HES6 | NR4A3 | NOTCH1 | NR4A2 |
| GLI1 | HSF2 | PHF5A | PCGF6 | SOX6 |
| HNF4A | JUN | STAT5A | PPARG | ZNF254 |
| HOXA3 | KLF6 | TAF4B | PRDM1 | ZNF91 |
| HOXA5 | LHX1 | TCF3 | RUNX1 |  |
| HSF4 | MAFA | TP63 | SOX7 |  |
| JUNB | MTA1 | TRIM22 | TBX20 |  |
| KLF6 | MXD1 | ZNF138 | TP73 |  |
| MAFA | MYT1L |  | ZNF268 |  |
| MXD1 | NR4A1 |  | ZSCAN29 |  |
| NOTCH1 | NR4A2 |  |  |  |
| NR4A3 | NR4A3 |  |  |  |
| PCGF6 | PHF5A |  |  |  |
| PHF5A | SOX6 |  |  |  |
| PPARG | STAT5A |  |  |  |
| PRDM1 | TAF4B |  |  |  |
| RUNX1 | TCF3 |  |  |  |
| SOX7 | TP63 |  |  |  |
| STAT5A | TRIM22 |  |  |  |
| TAF4B | ZNF138 |  |  |  |
| TBX20 | ZNF254 |  |  |  |
| TCF3 | ZNF91 |  |  |  |
| TP63 |  |  |  |  |
| TP73 |  |  |  |  |
| TRIM22 |  |  |  |  |
| ZNF138 |  |  |  |  |
| ZNF268 |  |  |  |  |
| ZSCAN29 |  |  |  |  |

**Table S9. The GO enrichment analysis of DEGs under oxidative stress in the category of biological process.**

**s**iCon_NT vs siCon_R0h:

| **Gene Ontology term** | **Cluster frequency** | **Genome frequency of use** | **Corrected P-value** |
| --- | --- | --- | --- |
| Response to corticosteroid stimulus | 5 out of 22 genes, 22.7% | 132 out of 14596 genes, 0.9% | 0.00034 |
| Response to inorganic substance | 6 out of 22 genes, 27.3% | 262 out of 14596 genes, 1.8% | 0.00049 |
| Response to steroid hormone stimulus | 6 out of 22 genes, 27.3% | 285 out of 14596 genes, 2.0% | 0.0008 |
| Response to reactive oxygen species | 4 out of 22 genes, 18.2% | 75 out of 14596 genes, 0.5% | 0.00117 |
| Response to metal ion | 5 out of 22 genes, 22.7% | 181 out of 14596 genes, 1.2% | 0.00164 |
| Response to stress | 11 out of 22 genes, 50.0% | 1660 out of 14596 genes, 11.4% | 0.00227 |
| Response to oxidative stress | 4 out of 22 genes, 18.2% | 119 out of 14596 genes, 0.8% | 0.00732 |
| Response to calcium ion | 2 out of 22 genes, 9.1% | 8 out of 14596 genes, 0.1% | 0.01612 |
| Response to hormone stimulus | 6 out of 22 genes, 27.3% | 510 out of 14596 genes, 3.5% | 0.02183 |
| Response to molecule of bacterial origin | 4 out of 22 genes, 18.2% | 158 out of 14596 genes, 1.1% | 0.02217 |
| Cell differentiation involved in embryonic placenta development | 2 out of 22 genes, 9.1% | 10 out of 14596 genes, 0.1% | 0.02586 |
| Embryonic placenta development | 2 out of 22 genes, 9.1% | 11 out of 14596 genes, 0.1% | 0.03158 |
| Response to external stimulus | 7 out of 22 genes, 31.8% | 810 out of 14596 genes, 5.5% | 0.03448 |
| Response to bacterium | 4 out of 22 genes, 18.2% | 181 out of 14596 genes, 1.2% | 0.03751 |

**siOXR1_NT vs siOXR1_R0h:**

| **Gene Ontology term** | **Cluster frequency** | **Genome frequency of use** | **Corrected P-value** |
| --- | --- | --- | --- |
| Regulation of cellular biosynthetic process | 18 out of 40 genes, 45.0% | 1524 out of 14596 genes, 10.4% | 9.88E-06 |
| Negative regulation of cellular process | 16 out of 40 genes, 40.0% | 1185 out of 14596 genes, 8.1% | 1.30E-05 |
| Regulation of biosynthetic process | 18 out of 40 genes, 45.0% | 1566 out of 14596 genes, 10.7% | 1.51E-05 |
| Regulation of cellular metabolic process | 21 out of 40 genes, 52.5% | 2260 out of 14596 genes, 15.5% | 2.45E-05 |
| Response to corticosteroid stimulus | 7 out of 40 genes, 17.5% | 132 out of 14596 genes, 0.9% | 2.57E-05 |
| Regulation of cellular process | 29 out of 40 genes, 72.5% | 4481 out of 14596 genes, 30.7% | 2.62E-05 |
| Regulation of nucleobase, nucleoside, nucleotide and nucleic acid metabolic process | 18 out of 40 genes, 45.0% | 1665 out of 14596 genes, 11.4% | 3.92E-05 |
| Regulation of macromolecule biosynthetic process | 17 out of 40 genes, 42.5% | 1468 out of 14596 genes, 10.1% | 3.93E-05 |
| Regulation of nitrogen compound metabolic process | 18 out of 40 genes, 45.0% | 1678 out of 14596 genes, 11.5% | 4.42E-05 |
| Regulation of macromolecule metabolic process | 19 out of 40 genes, 47.5% | 1910 out of 14596 genes, 13.1% | 5.38E-05 |
| Positive regulation of RNA metabolic process | 4 out of 40 genes, 10.0% | 18 out of 14596 genes, 0.1% | 6.04E-05 |
| Regulation of RNA metabolic process | 16 out of 40 genes, 40.0% | 1338 out of 14596 genes, 9.2% | 7.16E-05 |
| Regulation of metabolic process | 22 out of 40 genes, 55.0% | 2725 out of 14596 genes, 18.7% | 0.00012 |
| Regulation of cellular macromolecule biosynthetic process | 16 out of 40 genes, 40.0% | 1398 out of 14596 genes, 9.6% | 0.00013 |
| Negative regulation of biological process | 16 out of 40 genes, 40.0% | 1423 out of 14596 genes, 9.7% | 0.00016 |
| Response to inorganic substance | 8 out of 40 genes, 20.0% | 262 out of 14596 genes, 1.8% | 0.00019 |
| Response to abiotic stimulus | 9 out of 40 genes, 22.5% | 389 out of 14596 genes, 2.7% | 0.00034 |
| Regulation of transcription, DNA-dependent | 15 out of 40 genes, 37.5% | 1313 out of 14596 genes, 9.0% | 0.00036 |
| Regulation of transcription | 15 out of 40 genes, 37.5% | 1319 out of 14596 genes, 9.0% | 0.00038 |
| Response to calcium ion | 3 out of 40 genes, 7.5% | 8 out of 14596 genes, 0.1% | 0.00044 |
| Regulation of gene expression | 16 out of 40 genes, 40.0% | 1531 out of 14596 genes, 10.5% | 0.00045 |
| Regulation of primary metabolic process | 18 out of 40 genes, 45.0% | 2042 out of 14596 genes, 14.0% | 0.00085 |
| Regulation of biological process | 29 out of 40 genes, 72.5% | 5212 out of 14596 genes, 35.7% | 0.00097 |
| Response to stress | 16 out of 40 genes, 40.0% | 1660 out of 14596 genes, 11.4% | 0.00132 |
